# Regional differences, dynamic evolution, and influencing factors of high-quality medical resources in China’s ethnic minority areas

**DOI:** 10.3389/fpubh.2024.1436244

**Published:** 2024-09-06

**Authors:** Binghua Liang, Lifeng Huang, Zhuo Chen, Bangyan Hao, Chengcheng Li

**Affiliations:** ^1^School of Public Administration, Northwest University, Xian, Shanxi, China; ^2^School of Humanities and Social Sciences, Guangxi Medical University, Nanning, China; ^3^Department of Health Policy and Management, College of Public Health, University of Georgia, Athens, GA, United States; ^4^Humanities and Management School, Zhejiang Chinese Medical University, Hangzhou, China

**Keywords:** China, ethnic minority areas, health resource allocation, high-quality medical resources, dynamic evolution of resources

## Abstract

**Background:**

In China, as people’s standard of living improves and the medical service system becomes more sophisticated, the demand for higher-quality and improved healthcare services is steadily rising. Inequality in health resource allocation (HRA) is more pronounced in ethnic minority areas (EMAs) than in developed regions. However, little research exists on high-quality medical resources (HQMRs) in China’s EMAs. Hence, we examined the spatiotemporal dynamic evolution of HQMRs in China’s EMAs from 2007 to 2021 and identified the main factors affecting their respective HQMR levels.

**Methods:**

We selected tertiary hospitals to represent the quality of healthcare resources. We employed descriptive statistical techniques to analyze changes in the distribution of HQMRs from 2007 to 2021. We used the Dagum Gini coefficient and kernel density approach to analyze the dynamic evolution of HQMRs in China’s EMAs. We utilized the least squares dummy variable coefficient (LSDVC) to identify key factors affecting HQMR.

**Results:**

The number of HQMRs in each EMA has risen annually. The average number of tertiary hospitals increased from 175 in 2007 to 488 in 2021. The results of the Dagum Gini coefficient revealed that the differences in the HQMR level in China’s EMAs have slowly declined, and intra-regional disparities have now become the primary determining factor influencing overall variations. The kernel density plot indicated that the HQMR level improved significantly during the study period, but bifurcation became increasingly severe. Using the LSDVC for analysis, we found that gross domestic product (GDP) *per capita*, the size of the resident population, and the number of students enrolled in general higher education exhibited a significant negative correlation with HQMR levels, while GDP and urbanization rate had a significant promoting effect.

**Conclusion:**

The HQMR level in EMAs has risen rapidly but remains inadequate. The differences in HQMR between regions have continued to narrow, but serious bifurcation has occurred. Policymakers should consider economic growth, education, and population size rather than simply increasing the number of HQMRs everywhere.

## 1 Background

Health is a fundamental desire and basic necessity for all human beings (1), and health resources are the basis for promoting sustainable social development and maintaining people’s well-being (2). Medical resources constitute a crucial component of healthcare services and resources (3). Fair and effective allocation of medical resources is critical to promoting the expansion of public health (4). In the 2030 Agenda for Sustainable Development, the United Nations emphasized “ensuring universal access to health and healthcare services and achieving universal health coverage” as a key goal. At present, the unfair distribution of medical and health resources (MHRs) has become a global problem (5), especially in developing countries. For example, Hu et al. found that among the seven types of essential public services in China, the imbalance between the supply of and demand for medical and health services is recognizable (6).

Hence, the balance and sustainability of health resource allocation have attracted widespread attention in China. In 2009, China embarked on a new round of reforms of its medical and health system, and made substantial breakthroughs in solving the imbalance of health resource allocation and promoting the balanced distribution of regional health resources. However, with the improvement of living standards and the continuous improvement of the medical system, the existing health resources are no longer sufficient to meet the people’s diverse and multi-level demand for medical services. The people’s demand for high-quality medical resources (HQMR) is becoming increasingly urgent (7). However, owing to the limited level of economic growth and the uneven spatial distribution of health resources (7, 8), the disparity in medical services between urban and rural areas and across different regions continues to be prominent (8–11). As HQMRs cannot cover the entire population, People are compelled to relocate across regions to access higher-quality medical and healthcare services, which may exacerbate pre-existing social issues, increase the cost of healthcare access (12), and directly violate the right to health for some populations (13).

The balanced allocation of HQMRs has always been the focus of national healthcare. HQMRs function within a broader medical service network, including excellent medical talent, advanced medical techniques, first-rate instruments and equipment, and advanced medical information systems (14). In 2016, the Chinese government released the *Healthy China 2030 Plan*, which aims to “achieve a balanced distribution of high-quality medical care and health resources” (15). In March 2021, China published its *14^th^ Five-Year Plan for National Economic and Social Development* (2021–2025) as well as its *Outline of Long-term Goals*. A proposal has been put forth to expedite the expansion of HQMRs and to guarantee an equitable distribution of resources across various regions of the country (16). An investigation into regional differences in the level of high-quality medical treatment across China will help scholars to accurately understand the unbalanced allocation of HQMRs; such research is critical to advancing health system reforms.

At present, studies have fully recognized that the imbalance in the allocation of health resources is a serious health service challenge facing China, but most of them focus on the following aspects. First, it focuses on analyzing the geographical differences in the allocation of health resources in China from the horizontal perspective. In addition, Wan et al. used medical geography big data to analyze the spatial clustering patterns and spatial heterogeneity of medical resources in China (17). TongWang et al. evaluated the fairness of the allocation of medical and health resources in rural China from a macro perspective and compared the differences in fairness between the eastern, central and western regions in China (18). In addition, there are some non-state-level studies that analyze the inequality and disparity of health resource allocation in specific regions of China (19–22). The second is to analyze from a longitudinal dimension, focusing on the spatial–temporal evolution and sustainable development of health resource allocation (23). Shen et al. analyzed the spatial and temporal evolution trends and spatial agglomeration changes of medical and health resources in 41 cities in the Yangtze River Delta region of China from 2007 to 2019 (24). Chen et al. measured the level of medical and health service supply (MHSS) in 31 provinces in China from 2005 to 2020, and further studied the spatial distribution changes and dynamic evolution trends of MHSS (12). Overall, there have been many studies on the fairness of China’s health resource allocation, covering a wide range of dimensions. However, there have been few studies on regional differences in HQMR and the factors influencing them. Only a few scholars have studied China’s HQMR allocation and influencing factors at the national level, and there is a lack of systematic analysis of the fairness of HQMR allocation in specific regions (25).

Most of the recent related research has focused on China because China is a multi-ethnic country, with ethnic minorities concentrated along the borders and in remote parts of the country’s northwest and southwest regions, including Guizhou Province, Yunnan Province, Qingha Province, the Guangxi Zhuang Autonomous Region, the Ningxia Hui Autonomous Region, the Xinjiang Uyghur Autonomous Region, the Inner Mongolia Autonomous Region, and the Tibet Autonomous Region (26). Compared with the eastern and central regions, China’s ethnic minority areas (EMAs) are sparsely populated and underdeveloped in terms of economics and medical service provision. According to existing studies (27, 28), the quality, quantity, and fairness of health resources in EMAs are lower than those in other developed areas, and health resources and inequality persist (29), especially in terms of high-quality medical resources, there is still a large gap with other regions. This suggests that more attention should be paid to the specific situation and influencing factors of the allocation of HQMR in western China, in order to promote the expansion and sinking of HQMR in ethnic areas and promote the coordinated development of regional medical and health care. However, few studies have focused on the allocation of health resources in ethnic areas in China.

As far as we know, this is the first study on the dynamic evolution and influencing factors of the allocation of HQMR in ethnic areas. It can provide some inspiration for how to improve the allocation of HQMR in ethnic minority areas and other similar situations. The following are potential innovations from this study. First, this study uses the Dagum Gini coefficient and kernel density estimation to analyze the regional differences and dynamic evolution of the HQMR level in ethnic areas. These two methods utilize visualization techniques, such as three-dimensional graphs and line graphs, to more vividly and specifically illustrate the temporal and spatial evolution of health resources. Second, this study uses the LSDVC method to analyze the factors influencing the level of HQMR in ethnic areas, which can effectively solve the problem of endogeneity and is more suitable for the long panel data of this study. Finally, the analysis of influencing factors from multiple dimensions, such as economic development level, demographic characteristics, education level, and policy support, is of practical significance for guiding the government to implement precise policies.

## 2 Materials and methods

We relied on descriptive statistics to address changes in the HQMR distribution from 2007 to 2021. We used the Dagum Gini coefficient decomposition method to analyze regional differences in the HQMR distribution in EMAs, and visualized the distribution characteristics and evolutionary trends of HQMRs from 2007 to 2021 using kernel density estimation. We used MATLAB to draw a 3D kernel density map, and the least squares dummy variable coefficient (LSDVC) to identify key factors affecting the HQMR distribution.

### 2.1 Statistical methods

#### 2.1.1 The Dagum Gini coefficient

Decomposition methods are commonly used to measure regional differences, including the Theil index, the coefficient of variation, and the Gini coefficient; however, these approaches cannot further decompose regional disparities and compare the distribution of subsamples (30). The Dagum Gini coefficient can decompose a region into multiple sub-regions and calculate the total difference, intra-region difference, inter-region difference, and hypervariable density, thus breaking the above constraints more effectively. The specific equation of the Dagum Gini coefficient is as follows:


(1) $$G=\frac{\sum_{j=1}^{k}\sum_{h=1}^{k}\sum_{i=1}^{n_{j}}\sum_{r=1}^{n_{h}}|y_{ji}-y_{\mathrm{hr}}|}{2n^{2}\overset{↼}{y}}$$

(2) $${\overset{\leftarrow }{Y}}_{h}\leq \cdots {\overset{\leftarrow }{Y}}_{j}\leq \cdots {\overset{\leftarrow }{Y}}_{k}$$

In Equation 1, *G* denotes the overall Gini coefficient; *n* represents eight provinces categorized as EMAs; *k* refers to the number of secondary regions in the sample; *yji* (*yhr*) is the number of HQMRs in region *j* (*h*); $\bar{y}$ indicates the average value of an HQMR; *nj* (*nh*) is the number of provinces in region *j* (*h*); and *r* denotes the different provinces in region *j* (*h*). When the Gini coefficient is decomposed, the average of each region is sorted according to Equation 2.

According to the Dagum Gini coefficient decomposition method (31), the overall Gini coefficient *G* is divided into three parts: (1) the contribution of differences within regions *Gw*; (2) the contribution of differences between regions *Gnb*; and (3) the contribution of anti-variation intensity *Gt*, which satisfies *G = Gw + Gnb + Gt*. They are displayed in Equations 3–7:


(3) $$G_{w}=\sum_{j=1}^{k}G_{jj}p_{j}s_{j}$$

(4) $$G_{nb}=\sum_{j=2}^{k}\sum_{h=1}^{j-1}G_{jh}\left(p_{j}s_{h}+p_{h}s_{j}\right)D_{jh}$$

(5) $$G_{t}=\sum_{j=2}^{k}\sum_{h=1}^{j-1}G_{jh}\left(\begin{array}{c} p_{j}s_{h}+\\ p_{h}s_{j} \end{array}\right)\left(1-D_{jh}\right)$$

(6) $$G_{jj}=\frac{\sum_{i=1}^{n_{j}}\sum_{r=1}^{n_{j}}|y_{ji}-y_{\mathrm{hr}}|}{2n_{j}^{2}{\overset{↼}{y}}_{j}}$$

(7) $$G_{jh}=\frac{\sum_{i=1}^{n_{j}}\sum_{r=1}^{n_{h}}|y_{ji}-y_{\mathrm{hr}}|}{n_{j}n_{h}\left({\overset{↼}{y}}_{j}+{\overset{↼}{y}}_{h}\right)}$$

*Gjj* indicates the Gini coefficient in region *j* and *Gjh* denotes the inter-regional Gini coefficient in regions *j* and *h*. *Djh* is the relative influence between regions *i* and *j* using Equation 8.


(8) $$D_{jh}=\frac{d_{jh}-p_{jh}}{d_{jh}+p_{jh}}$$

The calculation formulas for *djh* and *pjh* are shown in Equations 9, 10, respectively, where *djh* is the difference in values between regions, and *Fh* (*Fj*) represents the cumulative density distribution function for region *j.* The equations read as follows:


(9) $$d_{jh}=\int_{0}^{\infty }dF_{j}\left(y\right)\int_{0}^{y}\left(y-x\right)dF_{h}\left(x\right)$$

(10) $$p_{jh}=\int_{0}^{\infty }dF_{h}\left(y\right)\int_{0}^{y}\left(y-x\right)dF_{j}\left(x\right)$$

#### 2.1.2 Nuclear density estimation

Kernel density estimation is an important non-parametric estimation technique that uses a smoothed peak function to fit the sample data as well as a continuous density curve to describe the distribution of random variables, which can reflect the distribution position, shape and ductility of random variables (32). The density function of random variable *X* is shown in Equation 11:


(11) $$f\left(x\right)=\frac{1}{Nh}\sum_{i=1}^{N}K\left(\frac{X_{i}-x}{h}\right)$$

*N* is the number of observations; *X* is the HQMR average; *K* is the kernel function; and *h* is the bandwidth. The kernel function does not greatly affect the shape of the curve, but it does affect the smoothness. The choice of bandwidth determines the shape of the curve. The larger the bandwidth, the smaller the variance of the kernel density estimate. In this study, we adopted a smoother Gaussian kernel and used kernel density estimation to explain the dynamic evolution of the HQMR distribution in EMAs from four perspectives (33): (1) distribution position; (2) shape; (3) ductility; and (4) polarization of the curve.

##### 2.1.2.1 Econometric dynamic panel model

In general, a generic panel model can be used, which can be divided into fixed effects (FEs) and random effects (REs). The specific formula is shown in Equation 12:


(12) $$Y_{\mathrm{it}}=\alpha +X_{\mathrm{it}}\beta +Z_{i}^{\prime }\delta +u_{i}+\varepsilon_{\mathrm{it}}\left(i=1,\;\cdots ,,;,,n;\;t=1,\;\cdots \;,T\right)$$

*Yit* is the number of HQMRs in EMAs; *Zi* is the individual feature that does not change with time; *Xit* is the individual and time-based change; *ui* is the individual effect; and *εit* is the residual disturbance term. If the *ui* is related to an explanatory variable, it is a FEs model. If the *ui* is independent of all explanatory variables, it is a REs model.

In many studies, the inertia of the dependent variable (34) is often ignored. If the lag term of the dependent variable is added as an explanatory variable. The model is shown in the Equation 13:


(13) $$Y_{\mathrm{it}}=\alpha +\rho Y_{i,t-1}+X_{\mathrm{it}}^{\prime }\beta +Z_{i}^{\prime }\delta +u_{i}+\varepsilon_{\mathrm{it}}\left(i=1,\;\cdots ,\;n;\;t=2,\;\cdots \;,\;T\right)$$

Using this dynamic panel model estimation will introduce dynamic panel bias, which is usually estimated by the difference GMM and the system GMM. We eliminated individual effects by creating first-order differences. The specific formula is shown in the Equation 14:


(14) $$\Delta Y_{\mathrm{it}}=\rho \;\Delta Y_{i,t-1}+\Delta \;X_{\mathrm{it}}^{\prime }\beta +\;\Delta \varepsilon_{\mathrm{it}}(i=1,\;\cdots \;,n;t=2,\;\cdots \;,T$$

When the sample’s features are small, the performance of the system GMM estimation is poor (i.e., the small-sample bias problem). Since the 15-year data selected for this study are long-term, the group is long-term and contains only eight provinces, and the panel size is small, estimation with systematic GMM regression may lead to small sample bias. Thus, we selected the bias-corrected least squares false variable (i.e., LSDVC), proposed by Bruno in 2005 (35), to accommodate unbalanced panels. The idea is to use the LSDVC to estimate the dynamic panel model as well as the bias of the LSDVC, and finally to subtract bias from the estimate of the LSDVC to achieve a consistent estimate (36). Based on prior research, we used the LSDVC estimator with FEs; it is stable in the presence of non-equilibrium panels, first-order sequence correlation, and set unobservable heterogeneity in the dynamic panel. We predicted the LSDVC estimator for initial dynamic panel estimation, which depends on the accuracy of N^−1^ T^−2^ and adopts the values of the system GMM estimate and the differential GMM estimate as the initial estimates.

### 2.2 Description of the data for the variables

We obtained data on the quality of medical resources and factors affecting the HQMR distribution in EMAs from 2007 to 2021 from the *China Health Statistics Yearbook*, published by the National Health Commission of China, and the *China Statistical Yearbook*, published by the National Bureau of Statistics.

Tertiary hospitals are the highest-ranking medical institutions in China’s existing medical service system; they tend to offer better medical services and management, medical quality and safety, an adequate technical level, and efficiency (37). Hence, we selected the number of tertiary hospitals in each province to represent the HQMR level.

The spatiotemporal variation of the HQMR distribution is a dynamic process supported by many factors. Based on relevant results (1–4), we mainly considered the impact of four elements: (1) demographic structure, (2) level of economic development, (3) education level, and (4) medical and health spending.

#### 2.2.1 Demographic structure

The size of the permanent resident population has a significant impact on the spatial distribution of HQMRs in China; hence, this variable was selected to represent the demographic structure. Gerdtham asserted that with an improved urbanization, more people could effectively receive high-quality medical and health services (38). In the present study, we used the urbanization rate to represent the level of urbanization.

#### 2.2.2 Level of economic development

This element significantly impacts the development of medical and health undertakings. We adopted GDP, and *per capita* GDP.

#### 2.2.3 Education level

There is a close link between residents’ education level and health status (39). The ultimate goal of the development of medical and health undertakings is to improve residents’ health. We used the number of students in colleges and universities per 100,000 people and the illiteracy rate to indicate education level. The illiteracy rate is inversely proportional, whereas the number of students in colleges and universities per 100,000 people is directly proportional to the education level.

#### 2.2.4 Government policy support

Government policy support plays a very important role in the allocation of medical resources. Intergovernmental transfer payments are mainly used to balance the differences in government revenue between regions due to differences in geographical environment or economic development levels. Government medical and health spending is crucial for measuring the development of medical and health undertakings. We use government transfer payments and government health expenditures to indicate the strength of policy support.

In sum, we selected nine indicators from the four elements (see Table 1 for details).

**Table 1:** Explanatory variables.

Factor	Specific index	Unit	Factor code
Demographic structure	Permanent population size	10,000 people	X1
	Population density	People/km^2^	X2
	Urbanization rate	%	X3
Economic development	local finance revenue in the general budget sector	Ten thousand yuan	X4
	GDP	Hundred million yuan	X5
	*Per capita* GDP	Ten thousand yuan	X6
Policy support	Government health spending	Ten thousand yuan	X7
Education level	Illiteracy rate	%	X8
	Number of students enrolled in regular higher education institutions per 100,000 people	People	X9

## 3 Results

### 3.1 The basic distribution of HQMRs in EMAs from 2007 to 2021

As seen in Table 2, the total amount of HQMRs in EMAs is denoted by a linear growth trend from 2007 to 2022. The number of tertiary hospitals rose from 175 in 2007 to 488 in 2021. The growth rate was 179%, with an average increase of 20.86 hospitals per year. Among them, the natural growth rate of Inner Mongolia and Guangxi was lower than the average of EMAs at 160 and 104%, this is mainly because it has a relatively high level of high-quality medical resources. The rest of the region was above average, and the highest was 750% in Tibet. In terms of stages, the number of tertiary medical institutions showed a slow growth or even negative growth trend during the period from 2007 to 2010. This period coincided with the initial stage of China’s medical reform, and the drastic fluctuations in the implementation of new policies led to fluctuations in the allocation of high-quality medical resources. During the period from 2010 to 201 During the period from 2010 to 2019, with the continuous improvement of medical reform policies, especially after the launch of the public hospital reform in 2010, the number of tertiary medical institutions showed a rapid upward trend, and the development trend was obvious. From 2020 to 2021, due to the impact of the epidemic, the growth rate of tertiary medical institutions slowed down, but it continued to increase. Overall, the level of high-quality medical resources allocation in ethnic minority areas showed a fluctuating upward trend.

**Table 2:** HQMR trends in EMAs from 2007 to 2022.

Province	2007	2012	2017	2021	Growth rate
Inner Mongolia Autonomous Region	35	33	67	91	160%
Guangxi Zhuang Autonomous Region	46	50	63	94	104%
Guizhou Province	22	27	49	79	259%
Yunnan Province	36	40	67	107	197%
Tibet Autonomous Region	2	2	7	17	750%
Qinghai Province	8	10	16	25	213%
Ningxia Hui Autonomous Region	6	4	13	19	217%
Xinjiang Uygur Autonomous Region	20	18	35	56	180%
EMA	175	184	317	488	179%

EMA, ethnic minority area.

Regarding the distribution of HQMRs in EMAs in 2021, there were 488 tertiary hospitals, averaging 61 per region. According to the geographic division, national areas are divided into two regions: northwest and southwest. The total amount of HQMRs in southwest China (297) was higher than that in northwest China (191). From a provincial perspective, Inner Mongolia, Guangxi, and Yunnan had more than 90 tertiary hospitals, with 91, 94, and 107, respectively. Four regions had a below-average number of tertiary hospitals, with Tibet and Ningxia having the fewest at 17 and 19, respectively. This is basically consistent with the ranking of economic development and population size in the provinces of ethnic areas, indicating that economic and demographic factors may have a greater impact on the level of high-quality medical resources and should be given priority consideration in future research. Further details are presented in Table 3.

**Table 3:** The HQMR distribution in China in 2021.

District	Province	Permanent population (10,000 people)	Total area (km^2^)	# of tertiary hospitals	Tertiary hospital	
					Per million people	Every 10,000 km^2^
Southwest	Guangxi Zhuang Autonomous Region	5,037	23.6	94	1.87	3.98
	Guizhou Province	3,852	17.6	79	2.05	4.49
	Yunnan Province	4,690	38.33	107	2.28	2.79
	Tibet Autonomous Region	366	122.8	17	4.64	0.14
	Southwest	13,945	202.33	297	2.13	1.47
Northwest	Inner Mongolia Autonomous Region	2,400	118.3	91	3.79	0.77
	Qinghai Province	594	72.23	25	4.21	0.35
	Ningxia Hui Autonomous Region	725	6.64	19	2.62	2.86
	Xinjiang Uygur Autonomous Region	2,589	166	56	2.16	0.34
	Northwest	6,308	363.17	191	3.03	0.53
EMA	Total	20,253	565.5	488	2.41	0.86

EMA, ethnic minority area.

In 2021, the average number of tertiary hospitals per million people in EMAs was 2.41. In terms of provinces, Xizang had the highest number of hospitals (4.64), while Guangxi had the fewest (1.87). In four regions (Guangxi, Guizhou, Yunnan, and Xinjiang), the number of tertiary hospitals per million people was lower than their average level in EMAs. In 2021, the average number of tertiary hospitals per 10,000 km^2^ in EMAs was 0.86. From a regional standpoint, the average level in the southwest (1.47) was much higher than that in the northwest (0.53), contrary to the outcome of the number of tertiary hospitals per million people. As for the number of tertiary hospitals per 10,000 km^2^, the top three provinces were Guangxi (3.98), Guizhou (4.49), and Ningxia (2.86), while for Tibet, Qinghai, and Xinjiang, this number was below the average level of hospitals in EMAs.

In summary, under the impetus of national policies, the overall level of high-quality medical resource allocation in ethnic minority areas is rapidly improving, but overall, development is still very uneven, and the total amount of high-quality medical resources in Tibet, Ningxia and other areas is relatively lacking. There is also a large gap with relatively economically developed areas such as Guangxi and Yunnan.

### 3.2 Dynamic evolution analysis

#### 3.2.1 Spatial difference analysis

**Figure 1:**
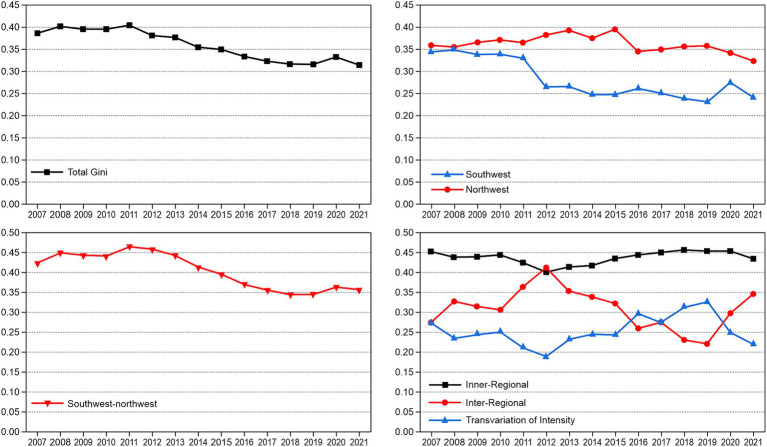
The variation and contribution rate of the HQMR level in EMAs.

We used the Dagum Gini coefficient decomposition method to measure the overall Gini coefficient, intra-regional Gini coefficient, inter-regional Gini coefficient, and contribution rate from 2007 to 2021 (see the Appendix for the results). Figure 1 depicts the trend of the Gini coefficient over the observation period, which indicates that the overall Gini coefficient in EMAs fell from 0.386 in 2007 to 0.314 in 2021, denoting an overall downward trend, with a total decline of 18.7% and an average annual decline of 1.2%. The total Gini coefficient increased from 2007 to 2011, reaching its highest value of 0.404 in 2011. The Gini coefficient revealed a downward trend after 2011. This suggests that the spatial distribution of HQMRs in EMAs tends to be rational.

Figure 1 depicts the changing trend of the Gini coefficients in the two EMAs from 2007 to 2021. The average Gini coefficients of the northwest and southwest were 0.281 and 0.362, indicating that the spatial distribution difference of HQMRs in the northwest was greater than that in the southwest. Although the Gini coefficient of the two regions had a certain fluctuation range, the overall trend was downward. Specifically, the Gini coefficients of the northwest and southwest fell from 0.359 and 0.344 in 2007 to 0.323 and 0.240 in 2021, respectively, with a total decline of 10 and 30.2%, respectively. This shows that HQMR optimisation in southwest China is better than that in northwest China.

Figure 1 presents the variation trend of the Gini coefficient differences between regions. The average Gini coefficient between the northwest and southwest was 0.404, and the difference was substantial. From the perspective of change, the regional differences between 2007 and 2021 demonstrated a narrowing trend, from 0.423 in 2007 to 0.356 in 2021, with an overall decrease of 15.8% and an average decline of 10.5%.

Figure 1 suggests that the contribution rate of each decomposition term to the overall Gini coefficient changed between 2007 and 2021. The contribution rates of intra-regional differences and super-variable density fell from 45.29 and 27.36% in 2007 to 43.39 and 22.02% in 2021, respectively. The contribution rates of intra-regional differences and super-variable density dropped from 45.29 and 27.36% in 2007 to 43.39 and 22.02% in 2021, respectively.

The contribution rate of regional differences rose significantly from 27.36% in 2007 to 34.58% in 2021. Intra-regional differences were the main source of overall differences. At the same time, with an increase in the volatility of the contribution rate of inter-regional differences, the contribution of inter-regional differences to overall differences increased.

#### 3.2.2 Kernel density estimation

**Figure 2:**
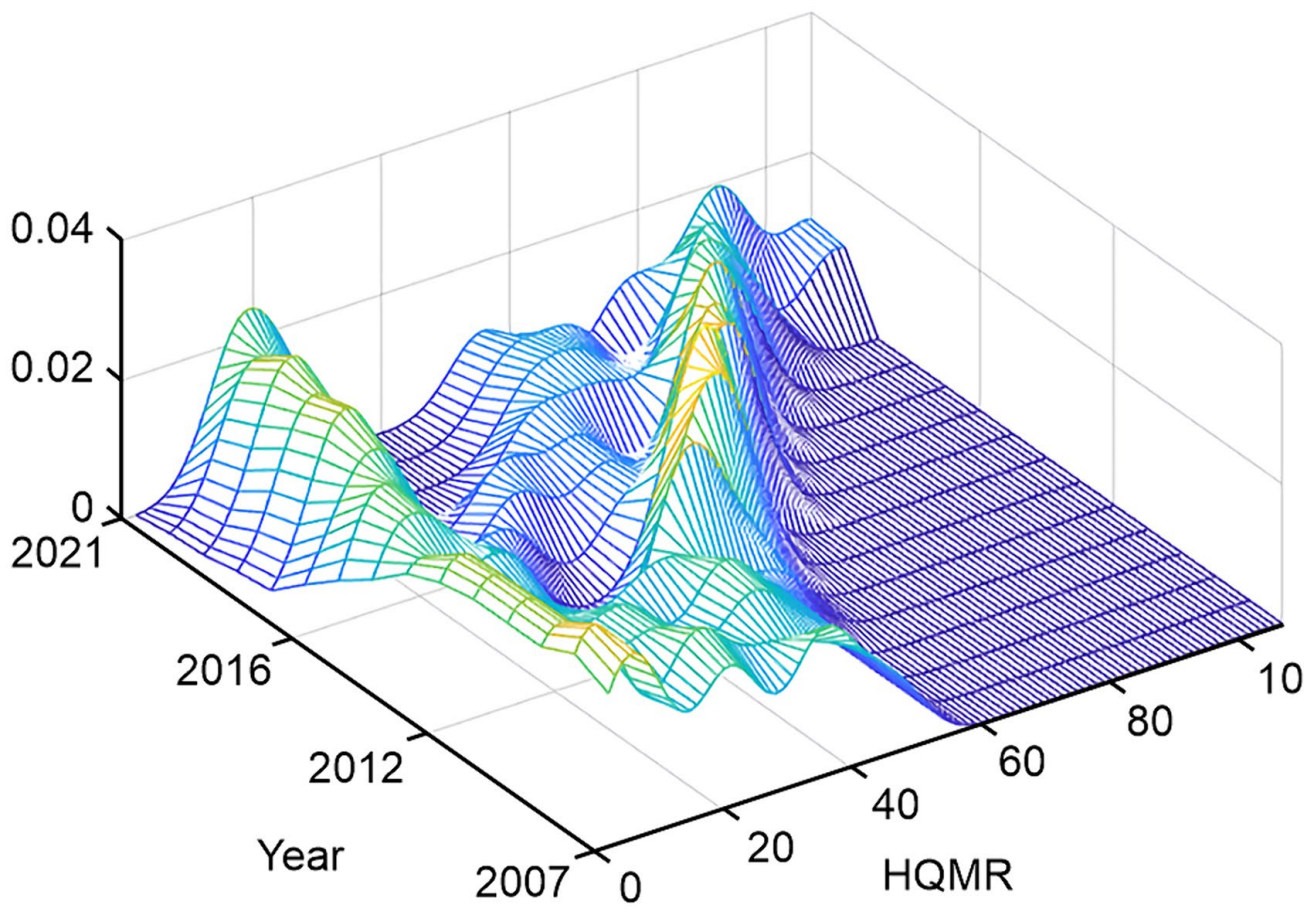
Estimate of nuclear density in EMAs.

To further study the dynamic evolutionary characteristics of the HQMR configuration level, we used the software MATLAB 2021a to estimate the kernel density. Figure 2 displays the results, specifically the dynamic evolution of the overall horizontal distribution of HQMRs in EMAs during the observation period. First, from the perspective of the distribution position, the focal point of the overall curve gradually shifted to the right, indicating that the number and level of HQMRs rose gradually. Second, from the distribution pattern, the main peak height of the overall curve first increased and then declined, and the width increased slightly, denoting that the absolute difference in each province had an expanding trend. That is, the HQMR level in each province was gradually dispersed, and the number of identities deviating from the mean increased gradually. Third, from the standpoint of distribution continuity, the distribution curve of EMAs presented a “right drag” phenomenon and slight widths, reflecting the slow widening of the gap between regional provinces, which is manifested as the gap between high-level regions (such as Yunnan and Guangxi) and low-level regions (such as Ningxia and Tibet). Finally, from the angle of differentiation, the curve as a whole changed from “unimodal” to “bimodal,” suggesting that the HQMR level presents a two-stage differentiation trend; this indicates a significant gradient effect in EMAs.

#### 3.2.3 Analysis of influencing factors of the HQMR configuration level

Given influencing factors and data types, we initially used ordinary least squares (OLS) regression and FE and RE models (in order to save space, the estimation results of OLS model, FE model and RE model are omitted). In building an OLS model, we found clear autocorrelations among the independent variables. According to previous studies, when autocorrelations exist among independent variables, GMM estimation can be adopted to solve the problem of estimation bias; however, these two methods are more suitable for short-term dynamic panels with *N*.

As seen in Table 4, the LSDVC-1 and LSDVC-2 models were generally consistent in the estimation outcomes, so we chose one of them for analysis. The system GMM has many advantages (e.g., good stability), and the estimation efficiency is higher than that of the differential GMM. Hence, we adopted the LSDVC-2 model and used the GMM estimator of the Blundell–Bond system to obtain the initial value.

**Table 4:** Analysis results of the LSDVC.

Variable	LSDVC-1	LSDVC-2
L1.y	0.141* (1.82)	0.182** (2.08)
X1	−2.353** (−2.24)	−2.719** (−2.09)
X2	0.0454 (0.32)	0.0256 (0.15)
X3	3.433** (2.00)	3.700* (1.93)
X4	1.331 (1.56)	1.348 (1.43)
X5	3.937*** (3.04)	4.336*** (2.75)
X6	−0.0000592*** (−3.76)	−0.0000627*** (−3.25)
X7	−0.185 (−0.35)	−0.252 (−0.40)
X8	0.0572 (0.05)	−0.0475 (0.03)
X9	−1.464*** (−5.68)	−1.551*** (−5.04)

LSDVC, least squares dummy variable coefficient; Z in brackets.

*p < 0.1, **p < 0.05, ***p < 0.01.

When we used the HQMR level of the first order as the independent variable, according to the estimation outcomes of the LSDVC-2 model, the HQMR level of the first order had a significant, positive correlation with the HQMRs of the current year. The estimated coefficient was 2.08, which passed the significance level test of 5%. The results show that HQMRs have obvious time inertia and a significant cyclic promotion effect.

The permanent population size was X1. According to the estimation outcomes of the LSDVC-2 model, there was a significant, negative correlation between the size of the permanent population and the HQMR level; the estimated coefficient was −2.09, which passed the significance level test of 5%. This suggests that HQMRs fall with a rise in the permanent population size.

As for X3, according to the estimation outcomes of the LSDVC-2 model, there was a significant, positive correlation between the urbanization rate and the HQMR level, with an estimated coefficient of 1.93, which passed the significance level test of 10%. This shows that with an improvement in the urbanization level, the HQMR level will improve.

X5 indicates GDP and X6 GDP *per capita*. According to the estimation outcomes of the LSDVC-2 model, both of them passed the significance level test of 1%. The estimated coefficients of GDP and *per capita* GDP were 2.75 and −3.25, respectively, implying that the improvement of the economic level has two sides to the enhancement of the HQMR level; On the whole, there is a positive correlation between the two, and there is a significant negative correlation when subdivided into individual levels.

The number of students in colleges and universities was X9. According to the estimation results of the LSDVC-2 model, there was a significant, negative correlation between the number of students in colleges and universities—representing 100,000 people per higher education institution—and the HQMR level. The estimated coefficient was −5.04. This suggests that as the number of college students increases, the level of HQMR will decrease.

The HQMR allocation levels from 2007 to 2021 generally resulted from a combination of factors. Among them, GDP *per capita*, the size of the permanent resident population, and the number of students in colleges and universities had a significant inhibitory effect, while GDP and urbanization rate had a significant promoting effect.

Population density (X2), transfer payments (X3), medical and health spending (X7), and the illiteracy rate (X8) did not pass the significance level test, implying that their effects on the HQMR levels were not significant.

In this section, we perform a robustness test to ensure whether the empirical results remain unchanged. We employ difference- and system-GMM estimators developed by Arellano and Bover and Blundell and Bond to determine the robustness of our results (40, 41). We check whether both difference- and system-GMM estimators are consistent by conducting two specification tests. First, we use the Sargan test of over-identifying restrictions, which identifies the overall validity of instruments. Second, we examine whether the second-order residuals are serially correlated. As a result, we find that the *p*-value in the Sargan test is not significant, which highlights that overall instruments are valid. Additionally, we confirm that second-order serial correlation is absent. Therefore, the inferences drawn by these estimations can be used in policy decision-making.

Table 5 reports the empirical results of the model. The system GMM estimation is used as an alternative analysis method. Therefore, the system GMM estimation supports our previous results obtained by the LSDVC method, which indicates that our empirical results are robust and constant. That is to say, the coefficients of the influencing factors affecting the HQMR level are still statistically significant and remain basically unchanged except for X1. X1 represents the permanent population size, easily Affected by slight sample deviation (31).

**Table 5:** Using alternative estimation.

Variable	Differential GMM	System GMM
L1.y	0.110* (1.78)	0.340** (2.15)
X1	−1.25 (−0.07)	1.843* (1.99)
X2	0.135 (1.15)	−0.487* (−2.82)
X3	4.48*** (3.57)	6.948*** (2.19)
X4	1.84** (2.56)	−0.116 (−0.11)
X5	4.304 (0.24)	4.081** (2.11)
X6	−4.312*** (−0.25)	−3.71* (−1.85)
X7	1.028** (2.63)	0.208 (0.23)
X8	0.0320 (0.05)	1.471 (0.51)
X9	−1.152*** (−11.08)	−1.460*** (−3.02)
Individual effects	Yes	Yes
Time effect	No	No
Constant	–	–
AR (1): *p*-value	0.002	0.021
AR (2): *p*-value	0.198	0.645
Hausman test	0.204	0.148
Observations	90	107

GMM, Gaussian mixture model; t in brackets.

*p < 0.1, **p < 0.05, ***p < 0.01.

## 4 Discussion

Focusing on EMAs in China, we investigated the temporal and spatial evolutionary trend of, and regional differences in, the HQMR level from 2007 to 2021, and we explored the influencing factors of the HQMR level in EMAs based on the four elements of demographic structure, level of economic development, education level, and medical and health spending. We drew four conclusions from the study results.

First, the total amount of HQMRs in EMAs is rising rapidly, but there is still a gap between EMAs and the rest of the country. Our results suggest that in the 15 years between 2007 and 2021, the number of tertiary hospitals expanded from 175 to 488, an increase of 179%. The average number of tertiary hospitals per million people rose from 0.93 to 2.41. The average number of tertiary hospitals per 10,000 km^2^ increased from 0.31 to 0.86. The growth rate of HQMRs in EMAs was significantly higher than the population growth rate, implying that the probability of residents obtaining HQMRs increased significantly (42). The results demonstrate that the Chinese government’s measures have achieved remarkable outcomes in enhancing the HQMR level in EMAs. However, compared with the relatively developed areas in the eastern and central regions, the allocation level of HQMRs in EMAs is still relatively insufficient, consistent with Yuan’s findings. Hence, to further realize the strategic goal of continuously improving the quality of health services and the level of health security, proposed in the *Healthy China 2023 Programme*, the government should strengthen its role in HRA, improve the feasibility of policies, reinforce relevant safeguards, ensure the implementation of pertinent policies, and increase the total amount of HQMRs in EMAs (43).

Second, we analyzed the regional differences and dynamic evolution of HQMRs in EMAs and found that the regional distribution of HQMRs tended to be reasonable; notwithstanding, the trend of the two-tier approach has become more serious.

Based on the Dagum Gini coefficient, the Gini coefficient of HQMRs in EMAs showed a downward trend during the observation period and an overall imbalance of HQMR allocation in EMAs. Our conclusions are similar to those of past studies on HRA in China (44, 45). From the perspective of inter-regional differences, internal differences in the northwest are greater than those in the southwest. Different provinces also have clear differences in location, resource endowment, economic scale, and financial capacity. The provinces and autonomous regions in the northwest are large in size, with many neighboring provinces (such as Inner Mongolia) bordering eight provinces and Qinghai bordering four provinces; hence, there are great differences between these provinces. The geographic location of each province in southwest China is relatively concentrated—the area is small, and development is relatively concentrated in all aspects. As a result, HQMR levels are more balanced in the southwest.

Regional differences have narrowed. The differences between the northwest and southwest revealed a narrowing trend, and the differences between regions declined. This is closely related to the western development policy put in place by the government. Since 1999, China has implemented the strategy of developing the western region, formulated a series of coordinated regional development strategies for 12 provincial-level administrative regions (including EMAs), and promoted the reform of the broader medical network, thus narrowing the gap in medical care among different provinces in the west. Intra-regional variation is the main factor of overall variation. From 2007 to 2021, the contribution rate of intra-regional differences was always greater than 40%, which was the main source of regional differences. Over time, the contribution rate of super-variable density gradually decreased, and the contribution rate of inter-regional difference gradually rose, indicating that the contribution of inter-regional differences to total differences slowly increased.

There was a particular gap in the overall HQMR distribution, showing a trend of two-level differentiation. According to the results of nuclear density estimation, the HQMR level in EMAs has significantly improved, and inter-regional differences have improved overall, but the gap between high-level and lower-level provinces still exists, and the polarization trend is becoming more serious. Simultaneously, there are regions with a very high HQMR level and regions with a very low HQMR level, and it is difficult to achieve the supply balance of HQMRs in the short term. As HQMRs are an important part of medical care resources, this distribution is similar to the distribution of overall medical resources in China.

Finally, the results of influencing factors indicate that the size of the permanent resident population, urbanization rate, GDP, *per capita* GDP, and the number of students in colleges and universities are key factors affecting the HQMR level. Cities with relatively small populations and fairly concentrated HQMRs have absolute advantages in terms of the HQMR level (46). With the increase in population size, the unbalanced allocation of medical resources is exacerbated (47), negatively affecting the improvement of the HQMR level. Moreover, the urbanization rate can improve the HQMR level. Urbanization has a significant effect on economic growth, especially in developing countries (48, 49). It is widely believed that a region’s economic development can provide strong support for healthcare spending, and governments in richer regions can afford to invest in HQMRs, whereas those in poorer regions cannot (50). Hence, urbanization will bring about economic growth in EMAs, thus promoting the improvement of the HQMR level. GDP and *per capita* GDP are important indicators reflecting the level of economic and social development, GDP positively promoted the HQMR level in EMAs, while *per capita* GDP had a negative inhibitory effect. On the one hand, with the increase in *per capita* GDP and the improvement of residents’ living standards, the demand for medical services has unreasonably increased, resulting in a decline in the efficiency of primary medical services (51, 52). The national policy focus is tilted toward primary medical institutions, which indirectly affects the improvement of the HQMR level; however, the current constraints on health spending have been relaxed, quickly leading to wasted allocated funds and lax supervision and management. In addition, the government pays no attention to the efficiency of the use of funds, which ultimately results in the inhibition of the positive promotion effect brought by total GDP growth and the impact on the HQMR level. The impact of education level on the HQMR level has been confirmed in previous studies. We further explored the impact of higher education level on the HQMR level and found that it had a significant negative impact on the HQMR level. Past studies have found that the overall mortality rate of Chinese residents decreased with an improvement in education level (53), and people with higher education tend to have better health awareness and living habits, thereby reducing their own risk of disease. As such, with the rise in the number of students in colleges and universities and the ongoing improvement of higher education institutions, the frequency and prevalence of medical treatment for residents have been reduced to a certain extent (54), thus reducing the demand for medical services and affecting the improvement of the HQMR level.

From an international perspective, this study has value. First, we recognize that residents’ health behaviors and HRA in China’s EMAs may be very different from those in other regions due to ethnic culture, folk medicine, geographic location, and other reasons (55). Therefore, we focused on the distribution of HQMRs in EMAs, providing research support for alleviating health inequality in EMAs and promoting the high-quality development of medical resources. Second, compared with past studies, we used the Dagum Gini coefficient and kernel density estimation to dynamically analyze the regional differences and evolutionary trends of HQMRs, which can more clearly show the developmental context of HQMRs. Our empirical results prove that there are significant differences in HQMR levels among EMAs and that they change over time. This suggests that spatial differences should be fully considered when studying the distribution of HQMRs in the future. Finally, we explored key factors affecting the HQMR level from multiple angles and found for the first time that population size, higher education level, and *per capita* GDP had a significant inhibitory effect on the HQMR level. In the future, coordination with the population level, education level, economic growth, and other dimensions should be considered when formulating relevant policies.

This study has a few limitations as well. First, we only measured the number of HQMRs by the number of tertiary hospitals and did not consider the number of beds or medical personnel in tertiary hospitals. Second, when exploring influencing factors, we found that factors such as the level of economic development, demographic structure, and education level significantly affected the improvement of the HQMR level in EMAs. The next step would be to further investigate critical factors affecting the distribution of HQMRs in EMAs from the structural perspective. At the same time, the shrinking and expansion of HQMRs should receive attention on the demand side of medical services. Therefore, future studies should collect demand-side data to construct a demand index and assess the spatiotemporal evolution and influencing factors of the demand for HQMRs in EMAs. Finally, due to data availability, we only discussed HQMRs at the provincial level; the situation in cities and counties requires further analysis.

## 5 Conclusion

We empirically examined the spatial and temporal trends in the HQMR level in EMAs and the factors influencing it based on the latest available data from official publications. This study provides insight into scientific management and guidance for policymakers to formulate effective policies for achieving higher levels of HQMR allocation. The main findings were that the overall HQMR level in EMAs is improving rapidly but is deficient in developed regions (16). Additionally, the differences in the regional distribution of HQMRs in EMAs are narrowing. However, they are still significant, and the severe polarization of resource allocation is a key issue that needs to be addressed in future research. At the same time, when formulating policies, it is necessary to take into account the need to coordinate with the level of economic development, the level of education, and the size of the population, rather than simply increasing the number of HQMRs. In summary, we recommend the following:

First of all, starting from the level of economic development, this study finds that the overall level of economic development has a significant positive impact on the allocation of high-quality medical resources, and *per capita* GDP has a negative impact on the allocation of high-quality medical resources. Therefore, the driving role of economic development in improving the allocation of high-quality medical resources should be given full play, and differentiated policies should be implemented according to different levels of economic development to achieve coordinated development of high-quality medical resources between the two subsystems. In areas with relatively good economic development, the construction of regional medical centers should be promoted, as should the development of high-tech research and development and the training of high-level health professionals, so that economic development can become a key driving force for improving the allocation of high-quality medical resources. In areas with relatively lagging economic development, the total amount of high-quality medical resources should be increased, the intensity of special intergovernmental transfer payments should be strengthened, and policies should be introduced to compensate for and introduce local social resources, enhance the government’s precision intervention, and guide the allocation of regional medical resources toward rationality.

Secondly, from the perspective of education level, this study found that higher education level has a negative impact on the level of high-quality medical resource allocation. As found in the previous study, this is mainly due to the higher health literacy of highly educated people. Therefore, on the one hand, we need to further improve the supply of high-quality medical resources in line with demand, meet the diverse, multi-faceted and multi-level medical service needs of different groups of people, and promote the overall improvement of the quality of high-quality medical resources. On the other hand, we need to incorporate theoretical ideas such as prevention-oriented and proactive medical care into the development of high-quality medical resources, not only to improve the level of disease treatment and optimize the allocation of hospital resources, but also to create a high-quality and efficient full-cycle medical and health service system.

Finally, in terms of population size, we must take into account the population situation in each region of the ethnic area, not just the total amount of high-quality medical resources, but also the *per capita* distribution and accessibility of the population. Therefore, in order to improve access to high-quality medical services for all citizens, it is recommended to accelerate the expansion and balanced distribution of HQMRs and promote coordinated regional development, rather than simply increasing the number of HQMRs.

In summary, this study is the first to comprehensively analyze the current situation of the allocation of high-quality medical resources in ethnic minority areas from the perspectives of spatiotemporal dynamic evolution and allocation influencing factors, and innovatively proposes policy recommendations for promoting the level of high-quality medical resources in different dimensions. This is of great practical significance for promoting the high-quality development of the medical and health service system in ethnic minority areas, and to a certain extent, it makes up for the research gap in this field.

## Data Availability

The original contributions presented in the study are included in the article/supplementary material, further inquiries can be directed to the corresponding author.
